# Importance of Mitochondria in Survival of *Cryptococcus neoformans* Under Low Oxygen Conditions and Tolerance to Cobalt Chloride

**DOI:** 10.1371/journal.ppat.1000155

**Published:** 2008-09-19

**Authors:** Susham S. Ingavale, Yun C. Chang, Hyeseung Lee, Carol M. McClelland, Madeline L. Leong, Kyung J. Kwon-Chung

**Affiliations:** Laboratory of Clinical Infectious Diseases, National Institute of Allergy and Infectious Diseases, National Institutes of Health, Bethesda, Maryland, United States of America; University of Wisconsin, Madison, United States of America

## Abstract

*Cryptococcus neoformans* is an environmental fungal pathogen that requires atmospheric levels of oxygen for optimal growth. For the fungus to be able to establish an infection, it must adapt to the low oxygen concentrations in the host environment compared to its natural habitat. In order to investigate the oxygen sensing mechanism in *C. neoformans*, we screened T-DNA insertional mutants for hypoxia-mimetic cobalt chloride (CoCl_2_)-sensitive mutants. All the CoCl_2_-sensitive mutants had a growth defect under low oxygen conditions at 37°C. The majority of mutants are compromised in their mitochondrial function, which is reflected by their reduced rate of respiration. Some of the mutants are also defective in mitochondrial membrane permeability, suggesting the importance of an intact respiratory system for survival under both high concentrations of CoCl_2_ as well as low oxygen conditions. In addition, the mutants tend to accumulate intracellular reactive oxygen species (ROS), and all mutants show sensitivity to various ROS generating chemicals. Gene expression analysis revealed the involvement of several pathways in response to cobalt chloride. Our findings indicate cobalt chloride sensitivity and/or sensitivity to low oxygen conditions are linked to mitochondrial function, sterol and iron homeostasis, ubiquitination, and the ability of cells to respond to ROS. These findings imply that multiple pathways are involved in oxygen sensing in *C. neoformans*.

## Author Summary

*Cryptococcus neoformans* is an obligate aerobic fungus that requires atmospheric levels of oxygen (21%) for optimal growth. However, the fungus is able to cause life-threatening brain infections in humans, where the oxygen tension is significantly lower than 21%. To understand the pathobiology of *Cryptococcus neoformans*, it is important to explore the molecular mechanisms adopted by the fungus to survive under low oxygen conditions. By using cobalt chloride, a hypoxia-mimicking agent, we isolated a number of mutants that are unable to grow in the presence of 0.7 mM CoCl_2_ as well as at low oxygen conditions. In this study, we show that mitochondria play an important role for *C. neoformans* to survive in low oxygen conditions. We demonstrate that mutants harboring mutations in the genes related to mitochondrial functions have mitochondrial membrane permeability defect and lowered respiration rate and are more sensitive to stress generating chemicals, in addition to their inability to survive at low oxygen conditions. Finally, we also show that when wild-type cells are exposed to hypoxia-mimicking cobalt chloride, expression of genes involved in respiration and iron and sterol homeostasis, as well as ubiquitination, changes significantly.

## Introduction

*Cryptococcus neoformans* is an opportunistic fungal pathogen that causes life-threatening meningoencephalitis primarily in immunocompromised patients [1]. *C. neoformans* is an obligate aerobe and its natural environment includes pigeon droppings, soil contaminated with avian guano [1] and decaying tree barks [2],[3]. In laboratory conditions, atmospheric levels of oxygen (∼21%) are required for optimal growth of *C. neoformans* and lower oxygen concentrations lead to a significant reduction in cell growth [4]. Upon inhalation, *C. neoformans* disseminates to central nervous system and causes life-threatening meningoencephalitis mostly in patients with immune deficiency. It is well known that oxygen concentrations in the human brain and other anatomical sites are significantly lower compared to atmospheric levels [5]. Thus, in order to establish infection in the brain, *C. neoformans* needs to sense and adapt to low oxygen conditions. Even though the mechanisms involved in oxygen sensing and adaptation to low oxygen conditions have been studied in humans and other organisms, this important aspect towards understanding the pathobiology of *C. neoformans* remains elusive.

In most eukaryotic organisms, molecular oxygen is essential for oxidative phosphorylation and biosynthetic processes. To survive in low oxygen conditions or hypoxia, organisms have evolved oxygen-sensing mechanisms that activate a complex set of responses. In mammals, a major effector of the hypoxic transcriptional response is the hypoxia inducible factor (HIF1α). Under high oxygen conditions, HIF1α is continuously degraded through hydroxylation while in low oxygen conditions, it is not hydroxylated thus avoiding degradation and activating the target genes [6]–[9].

In mammalian systems, cobalt chloride has been widely used as the hypoxia-mimicking agent. Studies done so far have shown that the CoCl_2_ mediated hypoxia-mimicking response is induced through the stabilization of HIF1α in the presence of oxygen [10]–[13]. The absence of *HIF1* homolog in *Saccharomyces cerevisiae* indicates a different mode of oxygen sensing [14]. While CoCl_2_ has been shown to have pleiotropic effects on cellular mechanisms in fungi, only a few of those have been linked to oxygen sensing [15]–[17].

Recent work from our laboratory has established a link between sterol synthesis, oxygen sensing and CoCl_2_ sensitivity in *C. neoformans* [18],[19]. Under low sterol or low oxygen conditions, *C. neoformans* homologs of the mammalian SREBP (sterol regulatory element-binding protein) transcription factor and its binding partner SCAP (SREBP cleavage-activating protein), named Sre1 and Scp1 respectively, regulate the expression of several genes involved in ergosterol biosynthesis and iron homeostasis. Mutations in *SRE1* and *SCP1* genes resulted in reduced growth under low oxygen condition and these mutants were not able to establish infection in the mouse brain [19]. Interestingly, both *sre1* and *scp1* mutants show reduced growth on media containing CoCl_2_. Further characterization of these mutants demonstrated that the response to CoCl_2_ in *C. neoformans* mimics certain aspects of the low oxygen condition by targeting enzymes in the sterol biosynthetic pathway [18].

In *C. neoformans*, apart from the genes involved in ergosterol biosynthesis, not many other genes have been identified that are required for adaptation to low oxygen as well as to high concentration of CoCl_2_. To identify genes involved in low oxygen response, our laboratory has taken two different approaches; 1) screening for the mutants directly under low oxygen conditions (manuscript in preparation) and 2) screening for the mutants that are sensitive to hypoxia-mimicking agent. In this study, we have explored the possibility of using CoCl_2_ as an effective chemical to screen for other factors involved in oxygen sensing. Using a genetic screening approach, we have isolated cobalt chloride hypersensitive mutants. Through characterization of these mutants, we have shown a link between the ability of cells to respond to CoCl_2_ and/or low oxygen conditions and mitochondrial function, sterol homeostasis, sensitivity to reactive oxygen species (ROS) and ubiquitination.

## Materials and Methods

### Strains, media, and growth conditions

*C. neoformans* serotype D genomic sequencing strain; B-3501A (http://www-sequence.stanford.edu/group/C.neoformans/index.html) was used as the wild type strain. All the strains in this study were derived from this strain. The strains were maintained on YES and YES+geneticin (100 µg/ml) where necessary. YES medium consists of 0.5% (w/v) yeast extract, 2% glucose and supplements containing uracil, adenine, leucine, histidine and lysine (225 µg/ml). For CoCl_2_ screening and sensitivity assays YES+0.7 mM CoCl_2_ was used. For all FACS experiments and O_2_ consumption assays yeast cells were grown to log phase (OD_600_ = 0.5) at 30°C in YES medium then 1 mM CoCl_2_ was added and grown further for 4 hrs at 30°C. Low-oxygen conditions (1% O_2_) were maintained using an Invivo_2_ 400 workstation (Ruskinn) at 37°C.

### Construction and screening of library for CoCl_2_-sensitive mutants

Since the frequency of random integration is very high by *Agrobacterium tumefaciens* mediated transformation (ATMT), a T-DNA insertion library of *C. neoformans* serotype D genomic strain (B-3501A) was made using ATMT [20]. The plasmid pYCC716 containing T-DNA fragment was used to create *A. tumefaciens* strain C603. This plasmid has a *NEO* gene conferring geneticin resistance. ATMT of *C. neoformans* was carried out as previously described [20]. To make a library, 30,000 individual transformants were picked and inoculated in 96 well plates containing YPD+50 µg/ml geneticin+200 µM cefotaxime. After 48 hr of growth at 30°C, glycerol stocks were made and stored at −80°C.

To identify the genes involved in the sensitivity to CoCl_2_, we screened T-DNA insertion library of *C. neoformans*. The primary screening for mutants sensitive to CoCl_2_ was carried out by replica spotting of the library consisting of 30,000 clones on YES+0.7 mM CoCl_2_ and YES+geneticin plates. Mutants that failed to grow on CoCl_2_ medium were selected for further analysis. A total 37 mutants (including 5 redundant isolates) were selected based on their sensitivity to CoCl_2_. Transformants selected for further analyses were streaked on selective YES+geneticin+cefotaxime medium.

### Mapping of T-DNA insertion site

Isolation and analysis of genomic DNA was carried out as described previously [21]. To identify the number of insertion events in these transformants, Southern hybridization was done using the ORF of the *NEO* gene as a probe. Radioactive probes were prepared using StripEZ kit (Ambion, Austin, TX) according to manufacturer's manual. By using vectorette system (Sigma, Woodlands, TX), T-DNA insertion site was mapped in *C. neoformans* mutants and genomic sequence flanking the insertion site was obtained. BLAST analysis of these sequences was done to reveal which loci have been affected.

### Construction of complementation strains

The wild-type gene for each affected gene was PCR amplified from B-3501A, cloned and sequenced. For complementation, using biolistic transformation method [22] T-DNA insertion mutants were transformed with the respective wild-type gene using *NAT* selection marker. Stable transformants were selected after repeated transfer on YES agar. PCR was used to identify integrative transformants containing an intact wild type gene and Southern blot analysis was used to confirm the transformation.

### Growth assays

Plate assays were carried out to determine if the mutants are sensitive to an external source of ROS-generating chemicals. YES plates containing 25 mM of sodium dihydrogen citrate, pH 4.0 (buffered YES) were made with 0.5 mM of H_2_O_2_ and 1mM of NaNO_2_. For other chemicals such as paraquat (0.25 mM) and diethyl maleate (2 mM), plates were made using regular YES. Cultures were either streaked or spotted and incubated at 30 and 37°C. For growth phenotype analysis serial dilutions of cultures were spotted on YES plates and incubated at 30°C, 37°C in ambient oxygen level and at 37°C under 1% O_2_ for 3–4 d.

### Confocal microscopy

Cells were grown overnight in YES medium, diluted in fresh YES to obtain initial OD_600_ of 0.2–0.3 and incubated for an additional 4 h. After 4 h, cells were harvested and resuspended in YES medium containing the MitoTracker Red CMXRos stain (Molecular probes, Eugene, OR) in the final concentration of 100 nM. Cells were incubated at 30°C for 1 h. After staining, cells were washed 3 times with 1× PBS and resuspended in 1× PBS. For mitochondrial DNA staining, cells were suspended in 10 mM HEPES buffer, pH 7.4, containing 5% glucose. SYTO 18 mitochondrial stain (Molecular probes, Eugene, OR) was added to a final concentration of 10 µM and cells were incubated at room temperature for 15–20 min. Before proceeding for microscopy, cells were washed and suspended in HEPES buffer. Images were collected on a Leica SP5 confocal microscope (Leica Microsystems, Exton, PA, USA) using a 100× oil immersion objective NA 1.4 zoom 4. Fluorochromes were excited using an Argon laser for SYTO18 and a 561 nm diode laser for MitoTracker Red CMXRos. Differential interference contrast (DIC) images were collected simultaneously with the fluorescence images using the transmitted light detector. Images were processed using Leica LAS-AF software (version 1.8.2 build 1465).

### ROS measurement

Cells were grown overnight in YES medium at 30°C, diluted in fresh YES and allowed to grow till OD_600_ reaches 0.4–0.5. Dichlorodihydrofluorescein diacetate (H2DCFDA) (Molecular Probes, Eugene, OR) at a final concentration of 10 µM was added for an additional 2 h to load the dye into cells. After 2 h, one set was supplemented with 1 mM CoCl_2_ and both sets were further incubated at 30°C for 4 h. Cells were harvested, washed 3 times with 1× PBS and an equal number of cells (2×10^6^) were resuspended in 1 ml of PBS. Fluorescence was then analyzed using a Becton- Dickinson FACScan Flow Cytometer. The parameters for Fluorescence-activated cell sorting (FACS) were set at excitation of 488 nm and for emission FITC channel using standard FL-1 filter (530/30 nm).

### Oxygen consumption assays

Cells were grown overnight in YES medium at 30°C, diluted in fresh YES to get starting OD_600_ of 0.2–0.3 and incubated for an additional 4 h. After 4 h, one set was supplemented with 1 mM CoCl_2_ and both sets were further incubated at 30°C for 4 h. Cells were harvested and washed with PBS and an equal number of cells (5×10^7^) were resuspended in 2 ml of YES and YES+CoCl_2_. Cells were then loaded into a sealed 2.0 ml glass chamber. Changes in oxygen tension were measured at room temperature with a Clark-type oxygen electrode, and the respiratory rate was calculated as a change in oxygen tension over time. In order to quantify mitochondrial respiration oxygen consumption, respiratory inhibitor Antimycin A (1 µg/ml) was used to block mitochondrial respiration.

### Gene expression profiling

Microarray slides were purchased from an academic consortium co-ordinated by T. Doering, C. Hull and J. Lodge at the University of Washington-St Louis. Arrays contain 7,738 70 bp DNA oligomers designed to uniquely recognize each gene in the *C. neoformans* serotype D genome plus control oligomers. Each oligomer is printed in duplicate. Overnight cultures of wild-type (B-3501A) strain was refreshed and grown in YES for 5 h and incubated for an additional 2 h in the presence or absence of 0.6 mM CoCl_2_. Wild-type strain does not show any growth inhibition at this concentration when treated only for 2 h. RNA was extracted from yeast cells using Trizol (Invitrogen, Carlsbad, CA), treated with RNAse-free DNase (Ambion, Austin, TX) for the removal of genomic DNA, and purified with RNeasy MinElute cleanup kit (Qiagen, Valencia, CA). RNA was labeled and hybridized as described before [18]. Data were collected using a GenePix 4000B scanner. For data analysis, features were examined and flagged using Genepix Pro 6.0 software (Axon Instruments, Foster City, CA) and confirmed manually. Data were further analyzed in mAdb database at http://mAdb.niaid.nih.gov (NIAID). Three biological repeats were performed using three independent RNA sets isolated from cells cultured on different days and the dye-reverse hybridizations were performed for all 3 sets. One set of RNA was also subjected to technical repeats. All statistically significant genes were identified by significance analysis of microarrays (SAM) using a mean false discovery rate (FDR) of 5% [23]. Only statistically significant genes were used for data analysis. To simplify presentation of the results, Figure 8 shows categorization of statistically significant genes identified by SAM, that have average changes greater than 2-fold. Table S1 lists the statistically significant genes identified by SAM that have average changes of greater than 2-fold in the wild-type strain upon CoCl_2_ treatment. All statistically significant genes identified by SAM are given in Table S2 in the supplementary material. The array data has been submitted at Gene Expression Omnibus (GEO) site (accession no. GSE11390).

## Results

### Construction of T-DNA insertional library and screening for cobalt chloride sensitive mutants

*Agrobacterium tumefaciens*–mediated transformation is a very useful technique to study *Cryptococcus*. The high frequency of random integrations by ATMT [20],[24] enabled the construction of a T-DNA insertional library using the *C. neoformans* serotype D genomic strain (B-3501A). The majority of transformants harbored a randomly integrated single copy of T-DNA and were mitotically stable (unpublished data). The library consists of 30,000 individual transformants.

**Figure 1:**
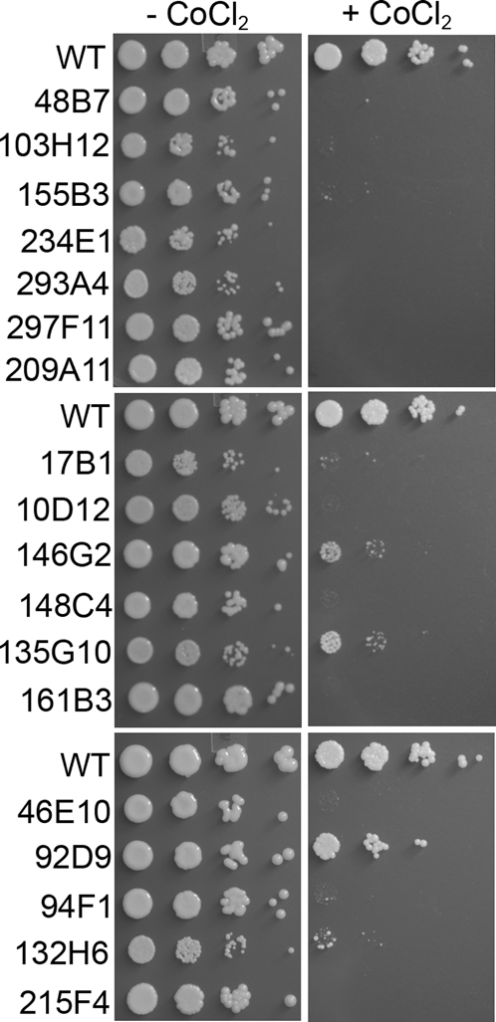
Cobalt chloride sensitive phenotype of T-DNA insertional mutants. Ten fold serial dilutions of wild type and T-DNA insertional mutant strains were spotted on YES and YES+CoCl_2_ plates and incubated at 30°C for 3 d.

**Figure 2:**
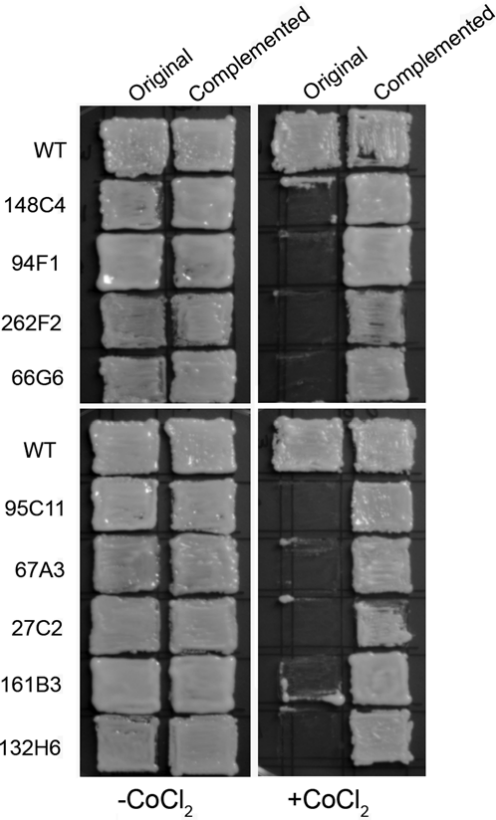
Complementation of CoCl_2_ sensitivity phenotype. Individual mutant was transformed with the corresponding wild type gene. Original and complemented strains were patched on YES and YES+CoCl_2_ plates and incubated at 30°C for 3 d.

To identify the genetic loci involved in the sensitivity to CoCl_2_, the T-DNA insertion library was screened for mutants sensitive to CoCl_2_. A total of 37 mutants that failed to grow on CoCl_2_ medium were selected for further analysis. Sequence analysis of the genomic sequence flanking the insertion site revealed that the T-DNA was inserted in a wide array of genes (Table 1). Based on the sensitivity towards CoCl_2_, mutants could generally be categorized into 2 types: mutants that either showed complete growth inhibition or those that exhibited partial growth on media containing 0.7 mM CoCl_2_ (Table 1). From the mutants listed in Table 1, mutants 146G2, 92D9, 135G10, 132H6, 17B1, 238D8, and 72B10 showed partial growth on medium containing cobalt chloride. All other mutants were hypersensitive to cobalt chloride and did not show any growth on CoCl_2_ containing plates (Table 1). Figure 1 shows the sensitivity pattern of some of these mutants from each group. To establish a link between a mutation and the CoCl_2_ sensitive growth phenotype, some of the mutants were complemented with wild-type gene. These complemented strains, when grown on CoCl_2_ containing plates, were no longer hypersensitive to CoCl_2_ and the growth was comparable to the wild-type strain (Figure 2). This indicates the defects in these genes result in the sensitivity towards cobalt chloride. In this screening, both *sre1* and *scp1* mutants were isolated several times and it has previously been demonstrated that the deletion of each of these two genes leads to CoCl_2_ sensitivity and these genes are involved in oxygen sensing in *C. neoformans* [18]. The repeated isolation of *sre1* and *scp1* mutants in the screen strongly attests to the suitability of this screening approach in the identification of genes responsible for growth under low oxygen conditions.

**Table 1:** A list of CoCl_2_ sensitive mutants isolated from T-DNA insertion library and their growth phenotypes.

Clone no.	Locus ID	Description^a^	Growth^b^ on					
			CoCl_2_	37°C/1%O_2_	H_2_O_2_	NaNO_2_	Paraquat	DEM
**Sterol Biosynthesis Pathway**								
10E11	CNC00950	SCAP^1^	**−**	**−/+**	**−**	**+/−**	**−/+**	**+**
87A1	CNJ02310	SREBP^1^	**−**	**−/+**	**−**	**−**	**+/−**	**+**
146G2	CNM00870	Squalene synthase	**−/+**	**−**	**−**	**−**	**−**	**−**
**Genes Involved in Mitochondrial Function/Energy Metabolism**								
10D12	CNC05260	H+ transporting ATP synthase	**−**	**−**	nd*	nd*	**−**	**−**
46E10	CNA00760	tRNA (guanine-N2-)-methyltransferase	**−**	**Ts**	**−/+**	**−**	**−**	**−**
66G6	CNC04010	HIG_1_N domain family	**−**	**Ts**	**−/+**	**−**	**−/+**	**−**
69G9	CNE00180	t-RNA Lysine	**−**	**−**	**−**	**−/+**	**−**	**−/+**
92D9	CNJ01940	Dihydrofolate reductase	**−/+**	**−**	**−**	**+/−**	**−**	**−/+**
135G10	CND04070	NADH∶ubiquinone oxidoreductase	**−/+**	**−**	**−**	**−**	**−**	**−**
161B3	CNM01080	ATP∶ADP antiporter	**−**	**−**	**−/+**	**+/−**	**+**	**+/−**
**Vesicular Transport**								
132H6	CNF00890	Importin beta-4 subunit	**−/+**	**Ts**	**−/+**	**−/+**	**−**	**−**
155B3	CNA06920	V ATPase subunit H	**−**	**−**	**−**	**−**	**−**	**−**
252C2	CNC07180	Vacuolar protein sorting 54	**−**	**−**	**−**	**−/+**	**−/+**	**+/−**
239E3	CNJ03270	Clathrin heavychain 1	**−**	**Ts**	**−**	**−**	**−**	**−**
**Regulatory Function Related**								
27C2	CNK03380	Fungal Zn(2)-Cys(6) domain family	**−**	**−**	**+/−**	**−/+**	**+**	**−**
215F4	CNF01510	Nonsense-mediated mRNA decay factor	**−**	**−**	**+**	**+**	**−/+**	**+/−**
238D8	CNN00160	Two-component protein-histidine kinase	**−/+**	**−/+**	**+/−**	**+**	**−/+**	**+**
293A4	CNM01040	C6 transcription factor	**−**	**−**	**−**	**+**	**+/−**	**−/+**
**Enzymes/Transporters**								
72B10	CNG01540	Hexose transport related protein	**−/+**	**Ts**	**−**	**−**	**−**	**−/+**
103H12	CNN01350	ATP dependent clp protease	**−**	**Ts**	**−**	**−**	**−**	**−**
148C4	CNF00750	Seroheme synthase	**−**	**−**	**−**	**−**	**−**	**+**
262F2	CNM00800	Amino acid transporter	**−**	**Ts**	**−**	**−**	**−**	**−**
297F11	CNF03950	Myo-inositol oxygenase	**−**	**−**	**−**	**+/−**	**+/−**	**+/−**
**Mutants with Multiple Insertion Sites** 2								
29A6	CNA07630	Hypothetical	**−**	**−**	nd*	nd*	**−**	**−**
94F1	CNH00220	Ubiquitin protein ligase	**−**	**Ts**	**−**	**−**	**−**	**−/+**
209A11	CNH03300	Microtubule binding protein	**−**	**−**	**−/+**	**−**	**+/−**	**−/+**
234E1	CNF03720	C-22 sterol desaturase	**−**	**−**	**−**	**−/+**	**−/+**	**−**
	CNG02960-	Hypothetical-						
	-CNG02950	-Dihydrodipicolinate synthetase						
**Hypothetical and Expressed Proteins**								
17B1	CNB03300	Hypothetical protein	**−/+**	**Ts**	nd*	nd*	**−**	**−**
48B7	CNL04460	Hypothetical	**−**	**−**	**−**	**−/+**	**−/+**	**+/−**
67A3	CNA00940	Expressed protein	**−**	**Ts**	**−/+**	**+**	**+/−**	**−**
95C11	CNF01170	Expressed protein	**−**	**−**	**−**	**−**	**+**	**−/+**
262A5	CNG02200	Hypothetical	**−**	**−**	**+/−**	**−**	**−/+**	**+/−**

a Annotations were obtained from NCBI database (http://www.ncbi.nlm.nih.gov) with additional hand editing based on BLAST searches.

b **+**, growth similar to WT; **+/−**, slight reduction in growth compared to WT; **−/+**, significant reduction in growth compared to WT; **−**, no growth. **Ts**, temperature sensitive, mutants did not grow at 37°C. ^*^, phenotype could not be determined because these mutants did not grow at pH 4.0.

1 Multiple mutants were obtained with same insertion sites.

2 Based on Southern analysis, these mutants have 2 or more T-DNA insertions. Only the loci that were able to produce PCR product and sequenced were shown.

### CoCl_2_-hypersensitive mutants are unable to grow in low oxygen conditions

**Figure 3:**
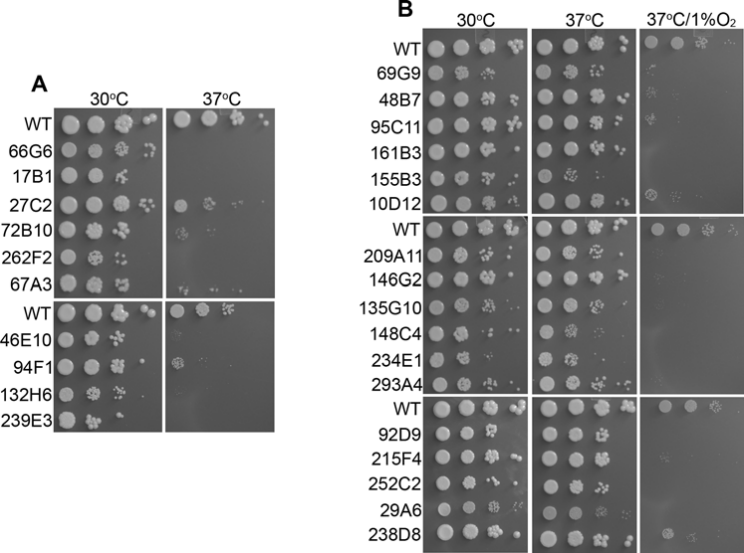
CoCl_2_ sensitive mutants exhibit hypoxia sensitive phenotype. (A). Mutants showing temperature sensitive phenotype. Yeast cells were serially diluted and spotted on YES and incubated at 30°C and 37°C for 3 d. (B). Mutants showing hypoxia sensitive phenotype. To check growth in low oxygen conditions, serial dilutions of wild type and mutant cells were spotted on YES and plates were incubated at 30°C, 37°C, and 37°C+1%O_2_ for 3 d.

To determine if the CoCl_2_ sensitive mutants also showed reduced growth in low oxygen conditions, cells were spotted on YES plates and incubated under different conditions: 30°C+ambient air (21% O_2_), 37°C+21% O_2_, and 37°C+1% O_2_. 37°C was chosen in this set of experiments in order to mimic the human body temperature. In the selected mutant pool, 10 of 32 mutants either failed to grow or grew poorly at 37°C, exhibiting a temperature-sensitive (Ts) phenotype (Figure 3A and Table 1). Most interestingly, the remaining non-Ts mutants showed reduced or no growth in low oxygen conditions (Figure 3B and Table 1). These results showed that screening for CoCl_2_ sensitivity is a useful approach to find mutants sensitive to low oxygen and suggested a strong correlation between CoCl_2_ sensitivity and sensitivity to low-oxygen conditions in *C. neoformans*. To identify the cellular processes involved in CoCl_2_ and/or low oxygen sensitivity, we categorized these mutants into different functional groups as shown in Table 1. Clearly, the processes involved in CoCl_2_ and/or low oxygen sensitivity are complex.

### Mitochondrial membrane permeability defect

**Figure 4:**
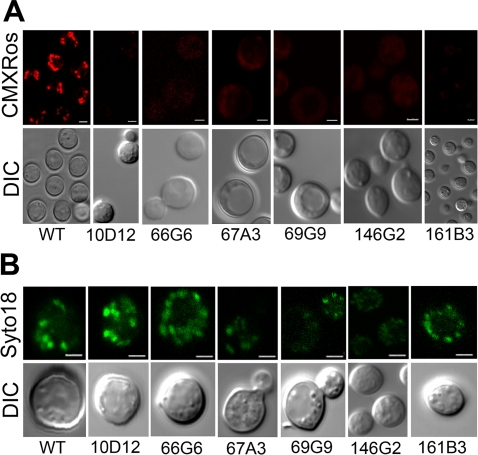
Confocal microscopy for mitochondrial staining. (A) Cells were grown to log phase and stained with mito-tracker dye CMXRos. Red color in the wild type shows the normal membrane permeability and diminished red color in the mutants suggested defect in mitochondrial membrane permeability. (B) Cells were grown to log phase and stained with SYTO18 dye for mitochondrial DNA. Green color indicates the presence of mitochondrial DNA. Experiments were done at least two times using mito-tracker red and SYTO18 dyes for these strains. Scale bar represents 3 µm.

We noted that seven mutants listed in Table 1 are directly related to mitochondrial function. In the mammalian system, cobalt chloride is known to affect mitochondrial function [11], [25]–[27]. Cobalt has also been shown to target mitochondria and induce respiratory deficiency in yeast [15],[17],[28],[29]. Various aspects of mitochondrial dysfunction can be detected by analyzing the mutants for perturbed mitochondrial membrane potential and efficiency of respiration. First, we examined all the cobalt chloride sensitive mutants for any perturbation in mitochondrial membrane potential by using mito-tracker dyes such as CMXRos. Confocal microscopy results showed that six different mutants were unable to retain the mito-tracker dye indicating the presence of dysfunctional mitochondria (Figure 4A, red stain). We observed heterogeneity in the cell size of these strains that maybe resulted from mutation caused by insertion of T-DNA. Four mutants belonged to the group involved in mitochondrial function in Table 1 (10D12, 161B3, 69G9, and 66G6). Both genes affected in 10D12 and 161B3 mutants have been studied in detail in *S. cerevisiae* [30]–[32]. Locus CNC05260 (mutant 10D12) encodes subunit f of the F(0) sector of mitochondrial F_1_F_0_ ATP synthase. The other locus, CNM01080 (mutant 161B3), encodes ATP∶ADP antiporter that catalyzes the exchange of ADP and ATP across the mitochondrial inner membrane. In the mutant 69G9, a tRNA lysine gene involved in mitochondrial protein synthesis [33],[34] is disrupted. The locus CNC04010 (mutant 66G6) encodes a hypothetical protein that has hypoxia induced protein conserved region (HIG_1_N domain). Two mutants (146G2 and 67A3) that also failed to accumulate the CMXRos dye did not belong to the group involved in mitochondrial function (Table 1). Mutant 146G2, containing a T-DNA insertion in *ERG9* gene encoding squalene synthase (CNM00870), also showed a defect in mitochondrial membrane potential. As the name suggests, this enzyme is involved in ergosterol biosynthesis and joins the two farnesyl pyrophosphate moieties to form squalene [35]. The hypothetical protein encoded by CNA00940 (mutant 67A3) has two *trans*-membrane domains and a weak homology to the t-RNA synthetase subunit. To determine whether these mutants lacked mitochondria, yeast mitochondrial DNA specific dye Syto18 was used for staining. Accumulation of Syto18 in the cells indicated that all 6 mutants contained mitochondrial DNA (Figure 4B, green stain). All the other mutants listed in Table 1 were also analyzed for reduced membrane potential by staining with CMXRos. The staining profile of these mutants was similar to that of wild type cells indicating that mitochondrial membrane potential was not disturbed in the rest of mutants (unpublished data).

### CoCl_2_-sensitive mutants have respiratory defects

**Figure 5:**
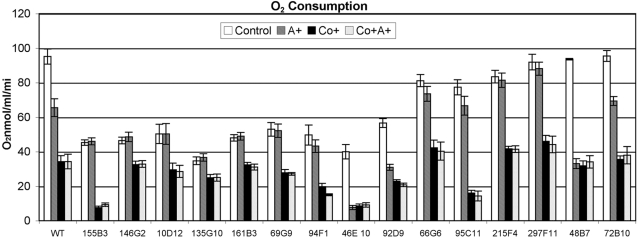
Oxygen consumption is reduced in yeast grown in CoCl_2_-containing medium. Yeast cells were grown as described in Materials and Methods. A total of 5×10^7^ washed cells were suspended in 2 ml media, and changes in oxygen tension were measured at room temperature using a Clark type electrode. Samples were measured with no addition of chemical (control), with antimycin A (A+), with CoCl_2_ (Co+), or with CoCl_2_ and antimycin A (Co+, A+).

As mentioned above, another way to analyze mitochondrial dysfunction is to assess the efficiency of respiration, which can be accomplished by measuring oxygen consumption. From the mutants listed in Table 1, all of the mutants in “mitochondrial function/energy metabolism” category and selected mutants from other categories were chosen to assay the rate of oxygen consumption. As presented in Figure 5, 9 mutants, 155B3, 146G2, 10D12, 135G10, 161B3, 69G9, 94F1, 46E10, and 92D9 showed a greater than 40% reduction in the rate of oxygen consumption compared to that of wild type in the absence of any chemical treatment. Four of these mutants, 146G2, 10D12, 161B3, and 69G9 also had a mitochondrial membrane potential defect as demonstrated in Figure 4. Even though mitochondrial membrane potential seemed unperturbed in the other 5 mutants, the reduction in oxygen consumption indicated those mutations seemed to have caused severe respiratory defects. Interestingly, mutant 66G6 had a mitochondrial membrane potential defect but showed only close to a 20% reduction in oxygen consumption rate compared to wild type in the absence of any chemical treatment. Among the rest of the mutants, for instance, 95C11 and 215F4 showed only a ∼15%–20% reduction while mutants 297F11, 48B7, and 72B10 had similar rates of oxygen consumption compared to that of the wild type.

To understand if the reduced oxygen consumption is due to a defect in the core respiration process, antimycin A was used. Antimycin A blocks mitochondrial respiration by inhibiting complex III of the electron transport chain and the use of antimycin A revealed the rate of oxygen consumption in the core respiration process. In wild-type cells, addition of antimycin A resulted in a 30% reduction in oxygen consumption. In mutants 155B3, 146G2, 10D12, 135G10, 161B3, and 69G9, the addition of antimycin A did not induce further reduction compared to the control samples (Figure 5). Similarly, mutants 94F1, 66G6, and 95C11 showed no significant difference in respiration rates between antimycin A treated and non-treated samples (*p*<0.02). These results suggest the reduction in the oxygen consumption in these mutants is due to the block in the antimycin A sensitive pathway and the genes targeted in these mutants are responsible for proper functioning of core respiration. It is not clear why in mutants 215F4 and 297F11 the oxygen consumption rate was close to the wild type and yet not sensitive to antimycin A treatment. In a second group of mutants, respiration was sensitive to antimycin A treatment. In the mutant 46E10 antimycin A caused 80% reduction in oxygen consumption while in mutants 72B10, 92D9, and 48B7 it was 30%, 40%, and 65%, respectively. This significant reduction in oxygen consumption in the presence of antimycin A in these 4 mutants indicates unlike above-mentioned mutants, cells are able to respire through the core electron transport chain.

Next, the cells were treated with cobalt chloride and assayed for oxygen consumption in the presence and absence of antimycin A. Compared to antimycin A, treatment with cobalt chloride showed an increased reduction in oxygen consumption in wild type. Addition of antimycin A to the cells grown in the presence of CoCl_2_ did not show any further reduction in the rate of oxygen consumption in the wild type as well as in all the mutants. This suggested that cobalt chloride strongly inhibits both antimycin A sensitive and antimycin A insensitive respiration pathways in all strains. Unlike in mutants where CoCl_2_ showed a significant effect on respiration compared to antimycin A alone, the reduction in the rate of respiration due to antimycin A or CoCl_2_ or in combination of both was similar in mutants 46E10 and 48B7. This indicates that in these mutants, antimycin A insensitive respiration pathways are more severely affected than the respiration through antimycin A sensitive core electron transport chain (ETC). These results indicate that 12 of the 15 genes affected in cobalt chloride–sensitive mutants influence oxygen consumption either through antimycin A sensitive mitochondrial respiration or antimycin A insensitive respiration.

### Mutants show elevated ROS levels and are sensitive to oxidants

**Figure 6:**
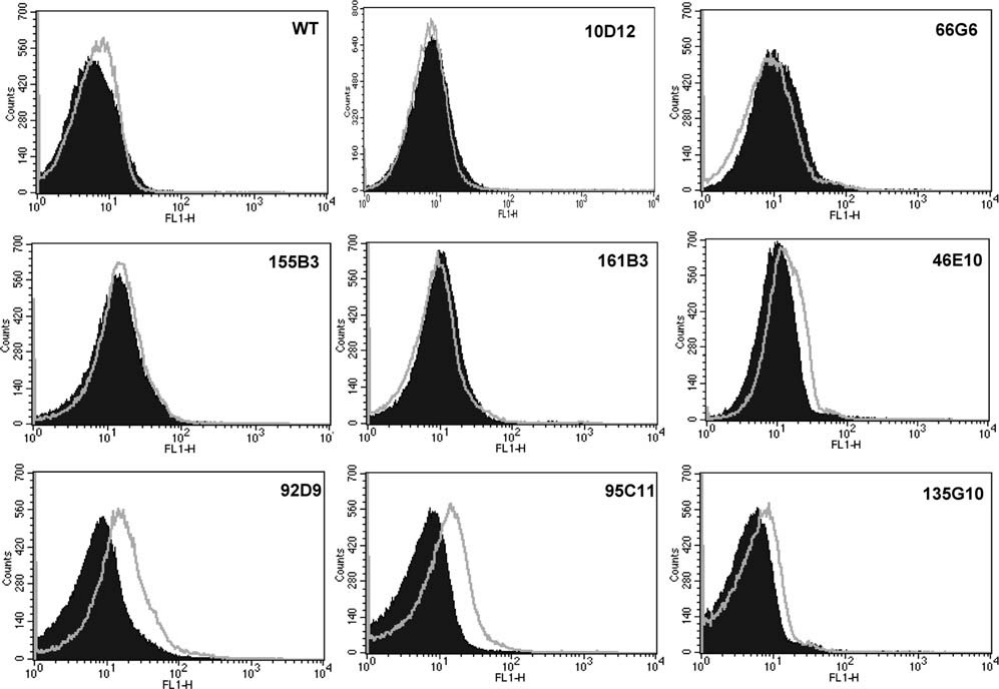
CoCl_2_-sensitive mutants accumulate reactive oxygen species (ROS). Wild-type and mutant cells were grown to log phase at 30°C, loaded with H2DCFDA and exposed to CoCl_2_ for 4 h. ROS accumulation was assessed by flow cytometry. Each panel represents the FACS analysis result of each strain; filled peaks (black) represent fluorescence intensity of untreated cells while line peaks (grey line) represent the fluorescence intensity of CoCl_2_-treated cells on the FL1-H scale. All the FACS experiments were done multiple times, and data presented here are a representative of the experiments.

Cobalt is one of the transition metals known to generate a spectrum of reactive oxygen species (ROS) in the Fenton reactions and subsequent lipid peroxidation [36],[37]. Generation and accumulation of ROS in the cellular environment due to cobalt chloride creates an imbalance in the oxidative state of the cells. This ROS-induced oxidative stress could be another factor that is leading to cobalt chloride sensitivity in the mutants. To investigate the possibility that the mutants have abnormal ROS production, the fluorescent dye H2DCFDA was employed. This probe increases its fluorescence when oxidized by ROS. Cells preloaded with H2DCFDA were treated with cobalt chloride and analyzed by FACS along with non-CoCl_2_–treated cells. Upon CoCl_2_ treatment, we observed only a slight increase in ROS levels in the wild type cells. One interesting observation to note here is that in four mutants, their intrinsic ROS levels were higher (1.5- to 2.3-fold increase in the fluorescence intensity) than that of wild type even without CoCl_2_ exposure (10D12, 66G6, 155B3, and 161B3; Figure 6). Compared to cobalt chloride treated wild-type cells, some mutants such as 46E10, 92D9, 95C11, and 135G10 showed an increase in ROS levels after incubation with CoCl_2_ (1.2- to 2.2-fold increase in fluorescence intensity; Figure 6). Therefore, most of the mutants have increased ROS levels either with or without CoCl_2_ treatment.

**Figure 7:**
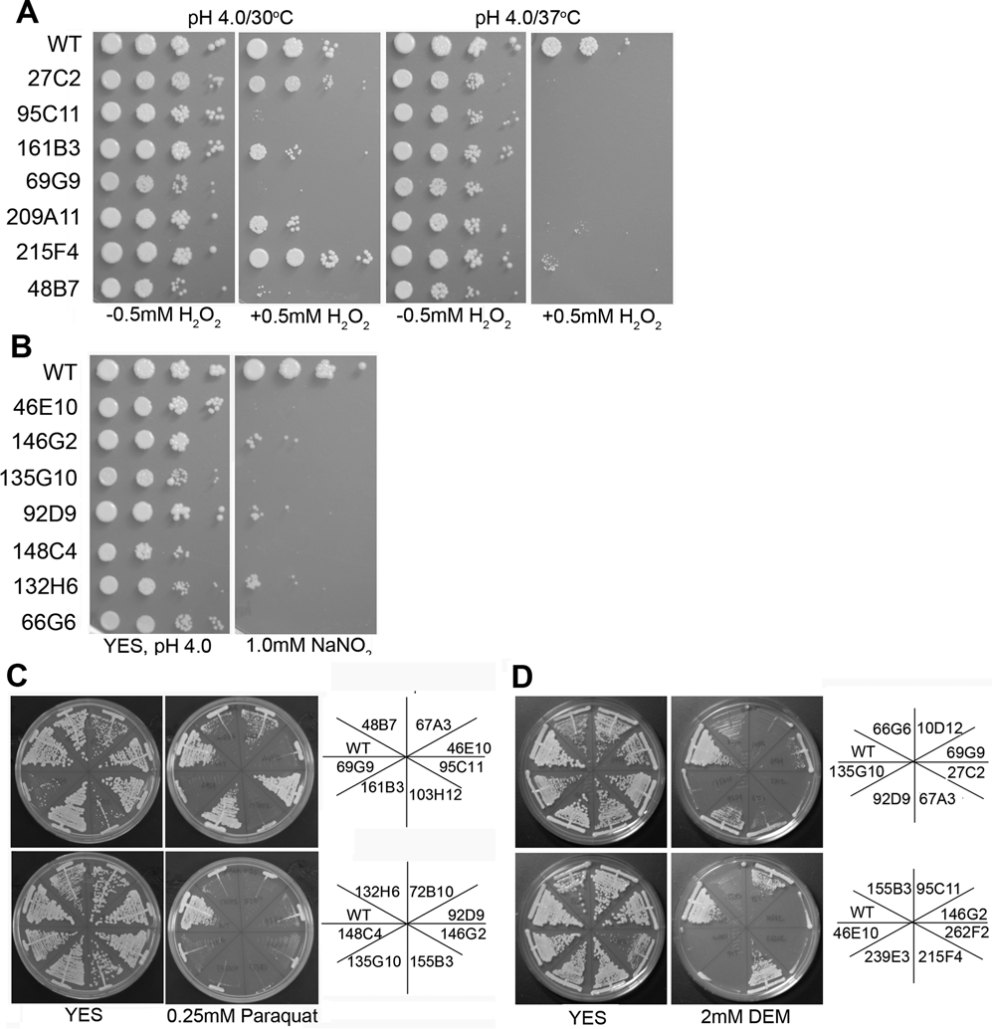
CoCl_2_-sensitive mutants are sensitive to ROS-generating reagents. Serial dilutions of yeast cells were spotted onto buffered, pH 4.0 YES with and without 0.5 mM H_2_O_2_ and incubated at 30°C and 37°C for 3 d (A), and with and without 1.0 mM NaNO_2_ and incubated at 30°C for 3 d (B). Wild-type and mutant strains were streaked on YES containing 0.25 mM paraquat (C) and YES+2 mM diethyl maleate plates (D). Plates were incubated at 30°C for 3 d.

Cellular antioxidant system influences the ability of cells to tolerate various oxidants. Unlike in wild-type cells, if the antioxidant system is not functioning properly in the mutants, they can exhibit hypersensitivity towards ROS and RNS (reactive nitrogen species) generating chemicals. To determine how CoCl_2_-sensitive mutants respond to the external source of ROS/RNS, growth was monitored on media containing H_2_O_2_, sodium nitrite, paraquat, and diethyl maleate. These four reagents act on different cellular targets and generate reactive oxygen/nitrogen species. H_2_O_2_ has the ability to directly damage nucleic acids, proteins, and lipids [38],[39]. The effect of H_2_O_2_ was analyzed at pH 4.0 and both 30°C and 37°C. 28 of the 29 mutants showed increased sensitivity towards H_2_O_2_ at 37°C compared to 30°C (Figure 7A and Table 1). Few mutants such as 95C11, 69G9, and 94F1 showed increased sensitivity even at 30°C. Sodium nitrite is known to generate nitric oxide (NO) at pH 4.0 [40],[41]. Nitric oxide reacts with different chemicals inside the cells forming reactive nitrogen species (RNS). The targets for RNS include protein-bound metal centers, thiols, DNA, lipids etc. [42]. NO has also been shown to inhibit complex III and complex IV of the respiratory chain [43],[44]. Based on plate assays, NO affected the growth in 25 out of 29 CoCl_2_-sensitive mutants (Figure 7B and Table 1). Paraquat (1,1′-dimethyl-4,4′-bipyridinium dichloride) is widely used to induce superoxide generation in cells. After its entry in mitochondria, paraquat is reduced by complex I leading to superoxide generation and subsequently extensive mitochondrial damage [45]. Figure 7C and Table 1 show that 28 of the 32 tested mutants are sensitive to paraquat. Diethyl maleate (DEM) is a glutathione-depleting agent and glutathione is one of the important non-enzymatic antioxidants that binds and inactivates ROS directly to protect cells against the toxic effects of ROS [46]. Exposure of cells to DEM leads to protein denaturation and increased intracellular ROS levels [47]. The majority of mutants tested (28 of the 32) showed sensitivity towards DEM (Figure 7D and Table 1). Furthermore, not all the mutants showed sensitivity to all four chemicals (Table 1). This was expected because based on the gene affected, different antioxidant systems would produce different outcomes in growth inhibition. Importantly, all the mutants showed sensitivity to at least one of the four ROS/RNS generating chemicals suggesting that all the CoCl_2_ sensitive mutants had a defect in handling the stress generated by ROS/RNS-producing reagents.

### Transcriptional profile of cobalt chloride treated wild-type cells

**Figure 8:**
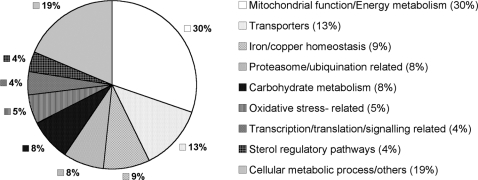
Pie chart. Pie chart shows the functional categories and distribution of the 182 genes whose expression has changed more than 2-fold in response to CoCl_2_. Annotations were obtained from the NCBI database (http://www.ncbi.nlm.nih.gov), with additional hand editing based on BLAST searches. Numbers in parentheses refer to the percentage of genes in each class and do not include the genes that are annotated hypothetical or expressed.

To gain an insight into the molecular basis for the effect of cobalt chloride treatment on *C. neoformans* cells, a whole genome microarray experiment was performed. Cells were harvested after 2 h of CoCl_2_ treatment. All genes with statistically significant differences in expression between wild type cells with CoCl_2_ and without CoCl_2_ were identified by significance analysis of microarray (SAM) using a mean false discovery rate (FDR) of less than 5% as described in Experimental Procedures. Of the 6,660 detectable genes analyzed, 979 genes were identified as significant genes by a mean FDR of 5% with SAM analysis. Of the 979 genes, 314 genes were up- or down-regulated by at least 2-fold. 42% of these genes have been annotated as hypothetical or expressed genes. The remaining 182 genes were categorized into different groups based on their annotated functions and presented in the form of a pie chart (Figure 8). The complete list of 314 genes is provided in the supplementary material (Table S1).

As the figure suggests, genes involved in mitochondrial functions form a major category (30%). Upon examination of the transcriptional profile of genes involved in mitochondrial function, it is apparent that CoCl_2_ repressed the expression of genes involved in the electron transport chain. Table 2 lists the genes encoding ETC components (complex I through IV) that were down-regulated at least 2-fold upon cobalt chloride treatment. Along with ETC component genes, CoCl_2_ treatment also led to a significant reduction in the expression of the gene encoding alternative oxidase (*AOX1*). Alternative oxidase bypasses complex III and IV by carrying out ubiquinol oxidation and reduction of dioxygen to water [48],[49]. As shown above, the respiration rate was reduced by ∼65% when the wild-type cells were treated with CoCl_2_. The possible reason for this significant reduction may be in part due to the down-regulation of ETC genes as well as alternative oxidase in response to CoCl_2_.

**Table 2:** Effect of CoCl_2_ on expression of respiration-related genes.

Locus ID	Fold Change*		Description^a^	
**Complex I**				
CNH02730	−1.54		NADH-ubiquinone oxidoreductase (subunit D)	
CNB01310	−2.10		NADH-ubiquinone oxidoreductase (subunit G)	
CND04070	−1.93		NADH-ubiquinone oxidoreductase 51 kDa subunit (NuoF)	
CNF03360	−1.45		NADH-ubiquinone oxidoreductase 30.4 kDa subunit, putative	
CND01070	−1.61		NADH dehydrogenase (ubiquinone)	
CNC07090	−1.91		NADH dehydrogenase (subunit E) putative	
CNE03960	−1.62		NADH ubiquinone oxidoreductase (subunit NDUFA12) putative	
CNM01810	−1.69		ETC complex I subunit conserved region, putative	
CNM02270	−1.77		ETC complex I subunit conserved region, putative	
CNE02800	−1.70		NADH dehydrogenase (subunit B), putative	
CNH01030	−1.18		NADH dehydrogenase 10.5 K chain	
**Complex II**				
CNA03530	−1.80		Succinate dehydrogenase (ubiquinone) (Sdh3 subunit)	
CNG03480	−3.40		Succinate dehydrogenase iron-sulfur (Sdh2 subunit)	
CNI03270	−1.54		Succinate dehydrogenase flavoprotein subunit precursor (Sdh1)	
CNB00800	−2.01		Mitochondrial inner membrane protein (Sdh4 subunit)	
CNJ00140	−3.12		Succinate dehydrogenase/fumarate reductase, flavoprotein subunit	
CND02060	−1.57		Succinate-semialdehyde dehydrogenase I, GabD	
CNF03900	−1.32		Succinate-semialdehyde dehydrogenase I, Alddh	
CNA05580	−1.27		Complex I protein (LYR family)	
**Complex III**				
CNF00630	−1.40		Electron transporter, cytochrome *c*_1_	
CNH02740	−1.17		ubiquinol-cytochrome C reductase complex (Qcr9 subunit)	
CNG00860	−1.19		Mitochondrial processing peptidase beta (Cor1 subunit)	
CNB01620	−1.88		L-lactate dehydrogenase, cytochrome *b*_2_ (Cyb2)	
CND04430	−1.12		ubiquinol-cytochrome C reductase complex (Qcr7 subunit)	
CNF03560	−1.27		ubiquinol-cytochrome C reductase complex (Qcr6 subunit)	
**Cytochrome**				
CNA06950	−3.46		Electron carrier, Iso-1-cytochrome *c*, (Cyc1)	
**Other Enzymes**				
CNA01500	−2.35		Alternative oxidase 1 (Aox1)	
CND02080	−2.27		FMN-dependent dehydrogenase, putative	
CNA04420	−2.69		oxidoreductase (Dehydrogenases (flavoproteins), FixC)	
CND02030	−1.09		Methylmalonate-semialdehyde dehydrogenase [acylating], putative	
CNM01770	−1.56		Aconitate hydratase (AcnA_Mitochondrial)	
CNK02510	−1.34		uroporphyrinogen-III synthase, (HemD)	
CNI03590	−1.69		phosphoenolpyruvate carboxykinase	
CNB04260	−2.27		5-aminolevulinate synthase, (Hem1)	
**Alternative Respiration Related**				
CNI00360	2.06	NADH dehydrogenase, (FAD-containing subunit, classII)		
CND02280	1.43	oxidoreductase (aldehyde/oxo group of donors, NAD or NADP as acceptor)		
CNC06220	2.39	glycerate-and formate-dehydrogenase (NAD or NADP as acceptor)		
CNI02360	3.46	NADPH dehydrogenase 2 (Oye2)		
CNB03640	2.58	oxidoreductase (NADH dehydrogenase, FAD-containing subunit)		
CNM00370	1.13	Aryl-alcohol dehydrogenase		
CNA05000	1.63	NADH dehydrogenase (FAD-containing subunit, class II), putaive		
CND00720	1.76	NADH-flavin reductase, putative		
CND02380	1.63	NADPH dehydrogenase, putative		
CNC00200	1.12	Methylase in ubiquinone/menaquinone biosynthesis, putative (UbiE)		
CNA04340	1.81	Mitochondrial hypoxia responsive domain protein, putative		
CNE02820	2.62	Mitochondrial ATPase, putative (AFG1_ATPase)		
CNE03260	1.02	Transcriptional activator (Hap3)		

a Annotations were obtained from NCBI database (http://www.ncbi.nlm.nih.gov) with additional hand editing based on BLAST searches.

*: Fold change values have been presented as log_2_ values.

Along with the genes involved in mitochondrial function are other groups of genes required for ergosterol biosynthesis, iron/copper homeostasis, oxidative stress, proteasome/ubiquitination function, carbohydrate metabolism, various transporters, specific transcription/translation, and some cellular metabolic processes. These data suggest that CoCl_2_ can induce a wide range of response in *C. neoformans*.

## Discussion

This study investigated the role of CoCl_2_ as an effective chemical to screen for probable pathways involved in oxygen sensing in *C. neoformans*. We show that most of the CoCl_2_ sensitive mutants are also sensitive to low oxygen conditions. These mutants fall into various functional categories including; mitochondrial function, sterol biosynthesis, vesicular transport, carbohydrate metabolism, and other cellular functions. Based on the characterization, we report cobalt chloride sensitivity and/or sensitivity to low oxygen conditions are strongly influenced by mitochondrial function, ability of cells to deal with ROS production, ubiquitination, and sterol homeostasis. These data highlight the complex nature of CoCl_2_ hypersensitivity and/or oxygen sensing and adaptation process in *C. neoformans*.

Many mutants obtained in our CoCl_2_ sensitivity screen were unable to retain Mitotracker dye indicating defective membrane potential. Genes affected in 3 of these mutants (10D12, 161B3, and 146G2) are related to structural components of the mitochondrial membrane. These results suggested proper maintenance of mitochondrial membrane potential is essential for *C. neoformans* to tolerate the high concentration of CoCl_2_ and to adapt to low oxygen conditions.

Another major defect in many of our CoCl_2_ sensitive mutants is the impaired oxygen consumption. It is clear that CoCl_2_ not only affected antimycin A sensitive core respiration but also inhibited antimycin A insensitive alternative respiration. Importantly, many of the cobalt chloride sensitive mutants have a defect in oxygen consumption through either mode of respiration, core or alternative. Cobalt chloride has the ability to induce a respiratory deficiency in cells [15],[17]. In response to specific inhibitors and oxidative stress, fungi are known to utilize alternative components of the respiratory chain consisting of alternative NADH dehydrogenases to bypass complex I and an alternative oxidase to bypass complex III and IV [44]. Although we do not have mutants that belong to the alternative respiration pathway, we isolated a complex I NADH∶ubiquinone oxidoreductase mutant (135G10). In addition, four more mutants defective in mitochondrial membrane potential (146G2, 10D12, 69G9, and 161B3) also showed only antimycin insensitive respiration. These data indicate that the presence of an intact respiration system is required for *C. neoformans* to handle the high concentrations of CoCl_2_. If respiration is affected so severely in the presence of CoCl_2_, it is possible that CoCl_2_ treatment affects the expression of genes encoding respiration related proteins. As described in Table 2, expression of a wide array of genes for subunits in complex I through III along with other genes involved in aerobic metabolism (*FIXC*, *HEMD*, *HEM1*) were down-regulated upon CoCl_2_ treatment. In addition, our array data showed that the *AOX1* gene was significantly repressed, while 10 different alternative dehydrogenases that belong to class II NADH∶ubiquinone oxidoreductases were up-regulated in CoCl_2_ treated cells. Two of the oxidoreductases listed in Table 2, (CNA05000 and CNI02360) have been shown to be up-regulated in a *SRE1*-dependent manner under 1% oxygen [19]. Interestingly, in *S. cerevisiae* under normoxic conditions aerobic genes are needed to encode proteins involved in aerobic metabolism such as oxidative phosphorylation while in low oxygen conditions hypoxic response genes are induced so as to allow the cells to utilize oxygen efficiently. These gene products involved in oxygen utilizing pathways include alternate cytochrome subunits and oxidases, and enzymes such as reductases and desaturases for heme, sterol, and fatty acid biosynthesis [50]. Our data here indicate in *C. neoformans* CoCl_2_ affects the expression of genes that are partially overlapping with genes expressed in oxygen limiting conditions. Perhaps, by regulating the expression of these genes in response to CoCl_2_, the cells can utilize oxygen more efficiently.

Many of the mutants in this study showed sensitivity to various ROS producing agents and a change in the intracellular ROS levels either with or without CoCl_2_ treatment. This increase in ROS levels in the mutants as well as hypersensitivity towards oxidants could be the cumulative effect of mutations they are harboring and the presence of the ROS inducing chemicals in the environment. To eliminate ROS from the cellular environment, a number of antioxidant systems such as superoxide dismutase, catalase, thioredoxin, glutathione, mannitol are in play [51],[52]. The sensitivity towards ROS generating chemicals in the mutants indicates that either the antioxidant systems are not functioning properly in these strains or irrespective of functional antioxidant system, cells are unable to handle the elevated levels of ROS. There may be some correlation between the ability of various chemicals to increase ROS levels and their increased toxicity to a CoCl_2_ sensitive mutant. In *Saccharomyces cerevisiae*, mitochondrial function has been shown to be required for resistance to oxidative stress [53]. Hence, there is a possibility that some of the mutants are sensitive to oxidants due to defects in mitochondrial function and not due to the antioxidant status of the cell. The mitochondrial respiratory chain is the main site of cellular ROS production. It has been reported that disruption of the electron transport chain leads to an increase in ROS levels in mammals as well as in *S. cerevisiae* [54]–[56]. Among our category of mitochondrial function mutants, although 135G10, the complex I NADH∶ubiquinone oxidoreductase mutant did not show elevated levels of ROS in log phase cells, this strain displayed increased ROS levels upon CoCl_2_ treatment compared to the wild type. Furthermore, two strains with a mitochondrial membrane potential defect (10D12 and 161B3) show increased levels of ROS in log phase cells even in the absence of CoCl_2_. These data support the notion that a perturbance in mitochondrial function can lead to intracellular accumulation of reactive oxygen species. Mitochondrial ROS generated during hypoxia and in the presence of CoCl_2_ have been implicated in cellular oxygen sensing in metazoans as well as in *S. cerevisiae* suggesting a role of mitochondria in oxygen sensing [56]–[58]. Therefore, like in hypoxic conditions in other systems, it is probable that CoCl_2_ also induces mitochondrial ROS levels in *C. neoformans* leading to the cross talk between mitochondria and the nucleus, which ultimately results in changes of gene expression profile. Since the mutants showing abnormal ROS levels are also sensitive to low oxygen conditions, it is possible that in oxygen depriving conditions, due to a mutation in a respective gene in the mutant, signals transduced through ROS are not able to activate a cascade of genes that sustain growth under low oxygen conditions.

Ubiquitination has been shown to be involved in targeting of nuclear-encoded pre-proteins into mitochondria, intracellular localization of macromolecules and degradation of superfluous/denatured proteins [59],[60]. In *S. cerevisiae*, in addition to these general functions, functional proteasomes and ubiquitin-dependent pathways are necessary for degradation of aerobic proteins (iso-1-cytochrome c) and modification of hypoxic proteins (Spt23, Mga2) [61],[62]. When *C. neoformans* cells were treated with CoCl_2_, we observed up-regulation of genes involved in proteasome function and ubiquitination suggesting similar mechanisms may be taking place (Table S1). In addition, complementation of clone 94F1 with CNH00220 locus encoding ubiquitin protein ligase rescued the CoCl_2_ sensitive phenotype (Figure 2), supporting the involvement of ubiquitination in handling the stress induced by cobalt chloride and/or low oxygen conditions in *C. neoformans*.

One of the early studies that suggested antagonism between iron and cobalt in fungal systems was carried out in the 1950s using *Neurospora crassa* as a model system [15]. Further studies in the same system showed growth inhibition due to cobalt could be reversed to some extent by iron [63] and cobalt induced iron deficiency resulted in the formation of siderochrome [16],[64]. In *S. cerevisiae*, even at sub-lethal cobalt concentrations, iron regulon is induced resulting in immediate expression of iron transporter genes to increase intracellular iron content. This response is similar to iron starvation conditions [65]. In mammalian systems, it is known that Co^2+^ can be substituted for the iron in porphyrin ring of oxygen-requiring enzymes leading to the lower affinity for oxygen and mimicking hypoxia environment [6],[66],[67]. In light of these studies in yeast, molds and mammals, gene expression profile in relation to iron/copper homeostasis made sense. Our data showed when wild-type cells are exposed to CoCl_2_, genes encoding high affinity iron permeases (*FRE3*, *CFT1*, *CFO1*, *CFT2*) as well as siderophore transporters (*SIT1*) were significantly up-regulated (Table S1), mimicking the iron deprivation response, although none of our CoCl_2_ mutants showed significant growth reduction in iron limiting conditions (unpublished data). As suggested in other systems, cobalt may also exert its antagonistic effects on iron metabolism in *C. neoformans* by competing with Fe^2+^ thereby affecting heme dependent and heme independent proteins. Transcription profile of cells treated with CoCl_2_ also revealed that genes involved in sterol regulatory pathways such as *ERG1*, *ERG3*, *ERG25*, *OLE1*, *NCP1*, *SCS7* are significantly induced (Table S1). These are oxygen-responsive genes and have been shown to be induced in low oxygen conditions in *C. neoformans* as well as in *S. cerevisiae* [19],[68]. Recent studies in *C. neoformans* showed the deletion of high affinity iron permeases (Cft1 and Cft2) leads to sterol synthesis defects [69]. Previous work from our laboratory identified an oxygen-sensing mechanism in *C. neoformans*, in which Sre1p and Scp1p are required for normal growth under low oxygen [19]. In our CoCl_2_ sensitive mutant screen, we identified two additional mutants related to sterol biosynthesis; squalene synthase (146G2) and C22-sterol desaturase (234E1), which are also sensitive to low oxygen conditions. Thus, in *C. neoformans* also iron-cobalt antagonism may disrupt iron and sterol homeostasis affecting the ability of cells to survive under high CoCl_2_ concentrations and/or low oxygen conditions.

Here, we have explored the possibility of using CoCl_2_ to understand the mechanism of adaptation to low oxygen conditions in *C. neoformans*. We propose CoCl_2_ may be mimicking hypoxia like conditions by inhibiting many of the mitochondria-related functions. These data have highlighted the complexity of oxygen sensing in *C. neoformans*. The unpublished data from our lab suggest involvement of a respiratory chain is only one aspect of oxygen sensing and there are multiple pathways involved in oxygen sensing which are not related to CoCl_2_ sensitivity (unpublished data). Further studies on the complex interactions between sterol and iron homeostasis, mitochondrial function and oxygen sensing, using this diverse set of CoCl_2_ sensitive mutants will shed light on the pathobiology of *C. neoformans*, especially in the brain environment.

## Supporting Information

Table S1 Gene expression upon 0.6 mM CoCl_2_ treatment.
(444 KB XLS)

Table S2 All statistically significant genes obtained by SAM analysis that were affected by 0.6 mM CoCl_2_ in wild type.
(78 KB XLS)
